# Editorial note

**DOI:** 10.2807/1560-7917.ES.2020.25.48.2012031

**Published:** 2020-12-03

**Authors:** Eurosurveillance editorial team

**Affiliations:** 1 European Centre for Disease Prevention and Control (ECDC), Stockholm, Sweden

We have recently received correspondence regarding a paper published this year, questioning both the content and the editorial procedures used to evaluate the article prior to publication. We can assure our readers and authors that we take comments relating to scientific content, the processing of articles and editorial transparency seriously.

All articles published by the journal are peer-reviewed by at least two independent experts in the field (or at least one in the case of rapid communications). The article in question was also peer-reviewed by two experts on whose recommendation the decision to publish was made. 

*Eurosurveillance* is seeking further expert advice and discussing the current correspondence in detail. We will, according to our existing procedures, evaluate the claims and make a decision as soon as we have investigated in full. In the meantime, it would be unfair to all concerned to comment or discuss further until we have looked at all the issues. 

Details on the editorial policies and processes are available here.

